# Iron stored in ferritin is chemically reduced in the presence of aggregating Aβ(1-42)

**DOI:** 10.1038/s41598-020-67117-z

**Published:** 2020-06-25

**Authors:** James Everett, Jake Brooks, Frederik Lermyte, Peter B. O’Connor, Peter J. Sadler, Jon Dobson, Joanna F. Collingwood, Neil D. Telling

**Affiliations:** 1 School of Pharmacy and Bioengineering, Keele University, Stoke-on-Trent, Staffordshire ST4 7QB United Kingdom; 2 School of Engineering, University of Warwick, Coventry, CV4 7AL United Kingdom; 3 Department of Chemistry, University of Warwick, Coventry, CV4 7AL United Kingdom; 4 J. Crayton Pruitt Family Department of Biomedical Engineering & Department of Materials Science and Engineering, University of Florida, Gainesville, Florida 32611 United States; 5 Department of Materials Science and Engineering, University of Florida, Gainesville, Florida 32611 United States

**Keywords:** Metals, Peptides, Dementia, Neurodegenerative diseases, Alzheimer's disease

## Abstract

Atypical low-oxidation-state iron phases in Alzheimer’s disease (AD) pathology are implicated in disease pathogenesis, as they may promote elevated redox activity and convey toxicity. However, the origin of low-oxidation-state iron and the pathways responsible for its formation and evolution remain unresolved. Here we investigate the interaction of the AD peptide β-amyloid (Aβ) with the iron storage protein ferritin, to establish whether interactions between these two species are a potential source of low-oxidation-state iron in AD. Using X-ray spectromicroscopy and electron microscopy we found that the co-aggregation of Aβ and ferritin resulted in the conversion of ferritin’s inert ferric core into more reactive low-oxidation-states. Such findings strongly implicate Aβ in the altered iron handling and increased oxidative stress observed in AD pathogenesis. These amyloid-associated iron phases have biomarker potential to assist with disease diagnosis and staging, and may act as targets for therapies designed to lower oxidative stress in AD tissue.

## Introduction

In the human brain, multiple functions are reliant upon iron as a co-factor, including, but not limited to, neurotransmitter synthesis, ATP production and myelin sheath formation^1,2^. As a result, a basal level of brain iron is required to maintain healthy tissues and brain iron homeostasis is strictly regulated^1–4^. Despite playing a vital role in the brain, iron can convey toxic effects when stored incorrectly or when it adopts abnormal chemical states^2,4–7^. The ability of iron to readily change oxidation state is an essential property required for its biological functionality. However, this ability also leads to its potential toxicity as it allows iron participation in Fenton chemistry, resulting in the production of reactive oxygen species (ROS) which are capable of inducing oxidative stress and neuronal injury^8–11^.

As a fundamental component of iron homeostasis, mammals store brain iron within the ferritin protein complex, in the form of a ferrihydrite-like mineral, (Fe^3+^)_2_O_3_•0.5H_2_O, a poorly crystalline ferric oxyhydroxide, typically accompanied by phosphate^1,12–14^. This form of storage prevents iron-associated ROS production, thereby protecting brain tissues from detrimental free radical burdens. Archetypal mammalian ferritin is comprised from two subunits: H-chain and L-chain chain ferritin, which assemble into a 24-subunit protein complex, ~450 kDa in molecular weight^1,13,15,16^. The complex is 12–13 nm in diameter, containing an 8 nm hollow core capable of storing up to 4500 Fe atoms^13^. Both H-ferritin and L-ferritin subunits have distinct functions that provide ferritin with an efficient means by which to store large numbers of iron atoms safely *in vivo*. H-ferritin (21 kDa) contains a di-iron binding site and possesses ferroxidase activity, catalysing the conversion of redox-active ferrous (Fe^2+^) iron into a redox-inactive ferric (Fe^3+^) state as it passes into the core. L-ferritin (19 kDa) does not possess any known enzymatic activity, but does contribute to long term iron storage, by accelerating the transfer of iron from the ferroxidase site to the ferritin core, whilst also stabilising ferritin’s complex structure^1,12,13^.

Evidence of disrupted iron homeostasis has been observed in the tissues of Alzheimer’s disease (AD) subjects, suggesting a link between altered iron handling in AD brain tissues and the development of the disorder^2,4,6,7,17–22^. AD is a progressive neurodegenerative disease that is the most common cause of dementia amongst the elderly and currently affects 5.4 million individuals in the USA alone^19,23–25^. The development of AD is heavily influenced by age, and due to increasing average life-expectancy, the number of AD cases is projected to nearly triple by 2050^25^. The underlying cause of AD is poorly understood and currently no cure exists.

Pathologically, AD is characterised by the presence of two hallmark lesions: intracellular neurofibrillary tangles comprised of hyperphosphorylated tau^26,27^, and extracellular plaques (also referred to as senile plaques) comprised primarily of the peptide β-amyloid (Aβ)^28–30^. Aβ is formed through cleavage of the amyloid precursor protein by secretase enzymes, resulting in the formation of multiple peptide isoforms varying in amino acid length^28,31–37^. The 42-amino acid isoform of the peptide is the most fibrillogenic, and is consequently most associated with AD traits, such as senile plaques and neurotoxicity^38^. Although the extent to which Aβ contributes to the pathogenesis of AD is a topic of much debate, even concerning whether deposition of Aβ is a physiological response as opposed to an inherently pathological occurrence^39^, it is commonly accepted that Aβ accumulation in the brain parenchyma is a fundamental event in the development of the disorder^28,31,40–42^.

Mounting evidence shows increased levels of iron, including iron minerals, to be present within tissue areas displaying AD pathology when compared to disease-free controls^20,21,43–51^. Iron minerals containing iron cations in a low oxidation state, such as magnetite (Fe_3_O_4_) and pure ferrous (Fe^2+^) minerals (e.g. wustite), as well as zero-oxidation-state iron (Fe^0^), are capable of catalysing Fenton redox chemistry, driving the production of ROS and potentially inducing oxidative stress. Such low-oxidation-state iron phases are inherently unstable and thus not expected to be preserved in biological systems. However, it has been suggested that ferritin malfunction and/or the interaction of Aβ with naturally occurring iron stores may result in chemically-reduced iron phases^43,45,47,50–54^. Indeed, ferritin has been shown to accumulate in Aβ plaque structures *in vivo*^55^, and increased levels of ferrous iron containing minerals have been recorded in pathological ferritin isolated from AD tissues compared to disease-free ferritin controls^50^.

Further evidence of iron accumulation within Aβ plaque structures has been demonstrated by HR-TEM where ferritin core-sized iron deposits with a crystal structure consistent with magnetite/maghemite were characterised embedded within extracted human amyloid plaque core material^47^; a result supported by more recent observations in other cases of Alzheimer’s disease^56^. Moreover, our recent synchrotron-based X-ray absorption studies have revealed the presence of nanoscale (*ca*. 200 nm) deposits of chemically-reduced iron within plaque structures located in the cortical tissue of APP/PS1 transgenic mice^57^, and amyloid plaque core material extracted from the grey matter in donated human brains from confirmed AD cases, including phases consistent with zero-oxidation-state iron in the latter^58^. These studies revealed dramatic nanoscale variations in iron oxidation state within *the same* amyloid plaque structures, highlighting the need for nanoscale resolution chemically-sensitive imaging in the investigation of metal biochemistry in living systems.

In addition, we previously demonstrated that the Aβ(1-42) peptide fragment is capable of chemically reducing unbound iron(III) oxyhydroxide and ferrihydrite to a pure ferrous phase *in vitro*^59,60^, whilst a recent *in vitro* spectrophotometric-based study has suggested that Aβ(1-40) can influence ferritin iron chemistry^61^. This evidence strongly implicates Aβ in the formation of chemically-reduced iron phases in AD.

As increased levels of oxidative stress are characteristic of AD pathogenesis^62–67^, chemically reduced, redox-active iron may represent a target for therapies intended to lower oxidative burdens, thereby inhibiting disease progression^68,69^. Furthermore, as iron redox chemistry has a profound effect upon its physical (particularly magnetic) properties, identifying iron phases specifically associated with Aβ pathology could provide a clinical biomarker for non-invasive disease diagnosis via magnetic resonance imaging (MRI)^70^.

Despite this growing body of evidence, the manner in which Aβ influences iron chemistry, even within the protein encapsulated core, and the chemical by-products formed through Aβ/ferritin interaction, remain unclear. Acknowledging both that ferritin is the primary form of iron storage in the brain^1^, and that Aβ/ferritin co-localises within AD tissues^55^, studying the chemistry of Aβ/ferritin interaction is vital to understand how altered iron homeostasis may contribute to the development of AD.

In this study we employed scanning transmission X-ray microscopy (STXM), a synchrotron-based X-ray spectromicroscopy technique, to examine the interaction of Aβ(1-42) with ferritin, and establish whether this interaction could result in the formation of the nanoscale, chemically-reduced iron phases observed within amyloid structures in the brain. STXM is a powerful technique that allows the chemical speciation of a sample to be determined to a spatial resolution of *ca*. 20 nm. The capacity for combined nanoscale spatial and chemical sensitivity, offered by STXM, is extremely important for investigations of amyloid/iron structures as the properties of inorganic inclusions have been shown to vary at the nanoscale.

Here, STXM was used to determine the distribution and chemical composition of protein and iron within *in vitro*-formed Aβ/ferritin aggregate structures at a nanoscale spatial resolution. We report a detailed characterisation of iron oxidation state formed through the interaction of Aβ and ferritin.

## Results

As multiple different laboratory-based and synchrotron experiments were performed for this study, it was necessary to use several different batches of the Aβ(1-42) peptide. For clarity, the different batches and associated investigations are explicitly listed in the Supplementary Information Table S1. The synchrotron work was performed using X-ray microscopes on two different synchrotron beamlines: PolLux at the Swiss Light Source; and I08 at the Diamond Light Source. As these instruments have slightly different performance characteristics (for example energy resolution), we have also stated in Table S1 which beamlines were used to obtain each dataset reported. All Aβ and ferritin incubations were performed under sterile conditions at 37 °C in a modified Krebs-Henseleit (KH) buffer medium (pH 7.4) containing physiologically relevant concentrations of Ca^2+^ and Mg^2+^ ^59,60^. Please refer to methods for further information regarding sample preparation/incubation.

### Aβ(1-42) incorporates ferritin into amyloid aggregate structures

**Figure 1:**
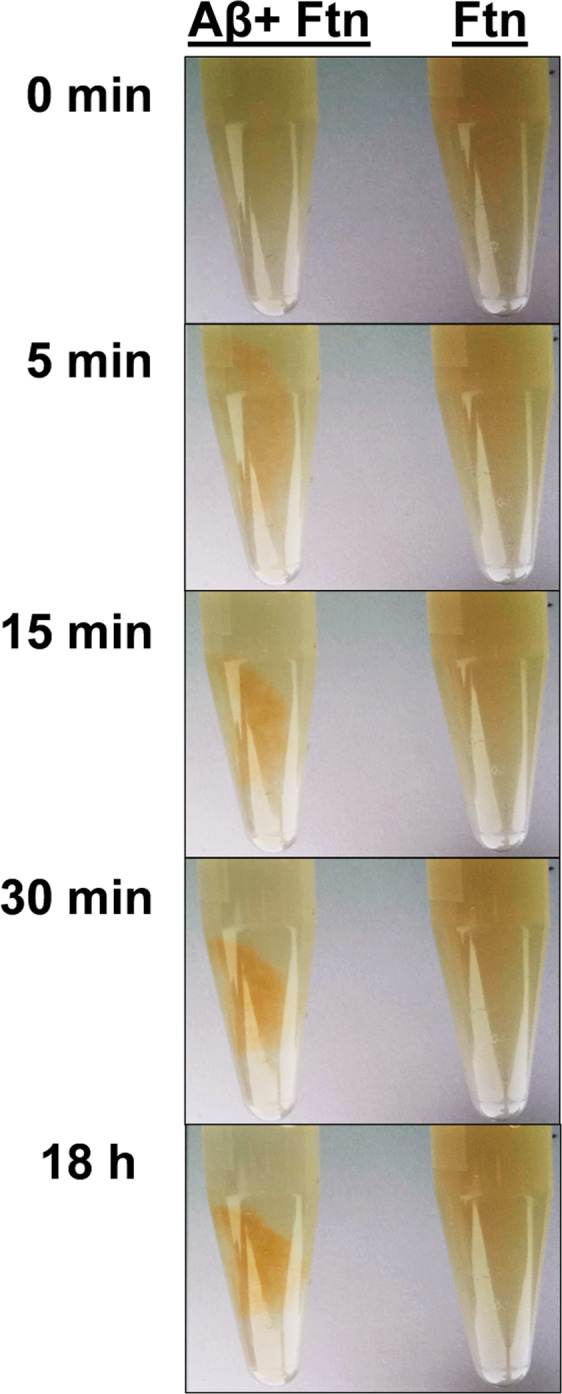
Time lapse images during incubation of ferritin and ferritin + Aβ. Images display ferritin (Ftn) incubated in the presence (left) and absence (right) of Aβ over an 18-hour period. Incubation times are shown to the left of each image.

Time-lapse images of ferritin incubated for 18 hours in the presence and absence of Aβ are displayed in Fig. 1. When incubated in the absence of Aβ, 1.3 µM ferritin formed a stable and uniform orange/brown colloidal dispersion that remained unchanged throughout the period of examination. Conversely when ferritin was incubated in the presence of 35 µM Aβ, dense orange precipitates formed after 15–20 minutes of interaction, suggesting the co-aggregation of Aβ and ferritin. Once formed, these precipitates remained unchanged over the 18 hours of imaging.

**Figure 2:**
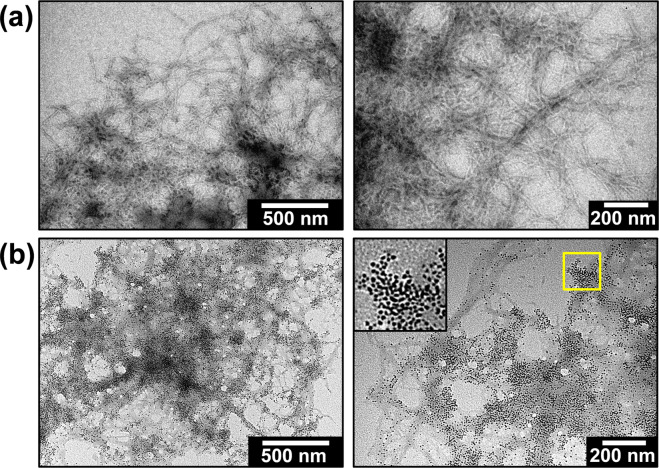
TEM images of Aβ/ferritin aggregate structures. Images display aggregates formed in **(a)** the absence and **(b)** the presence of ferritin (minimum 48 hours of co-incubation). Inset (bottom right) shows zoomed area highlighted in the yellow box.

TEM images of Aβ aggregate structures formed in the absence and presence of ferritin are displayed in Fig. 2. Where Aβ was incubated in the absence of ferritin, amyloid aggregates composed of well-defined fibrillar structures were observed (Fig. 2a), consistent in morphology with fibrillar amyloid structures reported previously^71^. However, when Aβ was incubated with ferritin, amyloid aggregates appeared to be composed of poorly defined structures containing a high concentration of electron dense fine particles (Fig. 2b). The average diameter (6.1 [±1.3] nm, n = 434) and morphology of these electron dense particles was similar to that of unstained ferritin standards imaged under TEM (6.8 nm [±1.1], n = 237); see Supplementary Fig. S1), where the electron dense regions correspond to ferritin’s iron oxide core. These TEM images suggest that Aβ had incorporated ferritin into its aggregate structures, complementing time lapse images of Aβ/ferritin suspensions where Aβ appeared to co-aggregate with ferritin (Fig. 1).

**Figure 3:**
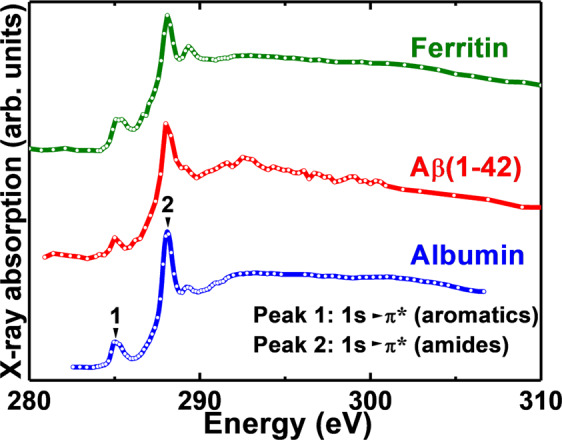
Carbon *K*-edge X-ray absorption spectra of albumin, Aβ(1-42) and ferritin.

X-ray spectromicroscopy (STXM) of Aβ structures formed in the absence of ferritin across the carbon *K*-absorption edge (280–320 eV) provided an X-ray absorption spectrum (Fig. 3; red) closely resembling an albumin protein reference (Fig. 3; blue). STXM examination of ferritin structures across the carbon *K*-edge generated an X-ray absorption spectrum (Fig. 3; green) similar to both albumin and Aβ. Whilst sufficiently different amino acids/peptides provide distinct carbon *K*-edge spectra (see for example Kaznacheyev *et al*., and Boese *et al*.^72,73^), the subtle differences between Aβ and ferritin spectra meant that measurements at the carbon *K*-edge alone could not be used to distinguish between the two species.

### Aβ/ferritin interaction leads to the chemical reduction of ferritin-stored iron

To confirm that the electron dense particles shown in Fig. 2b contained iron and to monitor the protein morphology and oxidation state of iron within Aβ/ferritin aggregates over an extended incubation period (144 hours), Aβ/ferritin aggregates were studied by STXM at the Swiss Light Source on the PolLux beamline.

**Figure 4:**
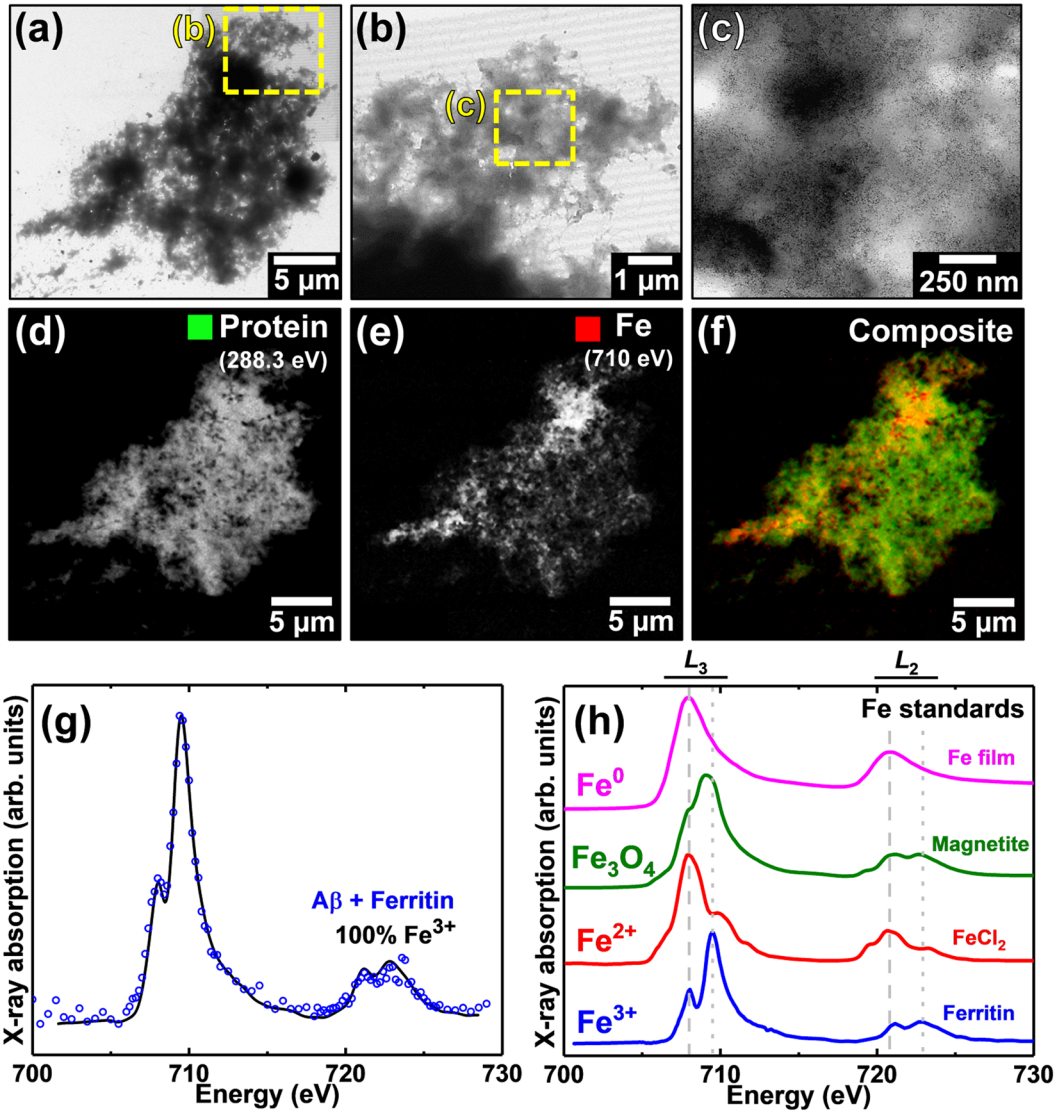
TEM and STXM analysis of an Aβ/ferritin aggregate formed following 48 hours of co-incubation. **(a-c)** TEM images of the Aβ/ferritin aggregate. Uniformly distributed electron dense fine particles can be seen in the highest magnification image in **(c)**. STXM speciation dependent maps **(d-f)** and an iron *L*_2,3_-edge X-ray absorption spectrum **(g)** from the aggregate. **(d)** Carbon *K*-edge protein map. **(e)** Iron *L*_3_-edge map. **(f)** Composite image displaying protein (green) and iron (red) content of the aggregate. **(g)** Iron *L*_2,3_-edge X-ray absorption spectrum from the iron content displayed in **(e)**. The solid line for the spectrum corresponds to the best fit curve created by superposition of suitably scaled iron reference X-ray absorption spectra. **(h)** Reference iron *L*_2,3_ edge X-ray absorption spectra for ferric (ferritin standard; blue), ferrous (FeCl_2_; red), magnetite (Fe_3_O_4_; green) and elemental (Fe^0^; magenta) iron phases. Grey dashed and dotted lines indicate the energy positions of the principal Fe^2+^ and Fe^3+^ absorption peaks respectively. See also Supplementary Fig. S3 for additional ferritin iron *L*_2,3_-edge X-ray absorption spectra.

Following 0.5 and 48 hours of Aβ/ferritin co-incubation, multiple protein dense aggregates were observed using STXM. A representative example of an aggregate structure formed following 48 hours of incubation is shown in Fig. 4. TEM imaging of the aggregate revealed a poorly defined fibrillar structure containing electron dense fine particles widely spread throughout the aggregate (Fig. 4a–c), indicating the iron oxide core of ferritin was preserved.

Speciation-dependent contrast maps of the aggregate collected at the carbon *K*-absorption edge (Fig. 4d) in order to observe the protein distribution, and the iron *L*_3_-absorption edge (Fig. 4e), showed iron to be distributed throughout the entire aggregate structure, demonstrating the co-aggregation of Aβ and ferritin. A similar distribution of iron was observed within an Aβ/ferritin aggregate formed following 0.5 hours of incubation, as displayed in Supplementary Fig. S2.

To establish the precise chemical nature of the iron within the aggregate shown in Fig. 4, STXM examination was performed across the entire iron *L*_2,3_-absorption edge (700–740 eV). A non-linear least-squares fitting procedure was employed for the iron *L*_2,3_-edge X-ray absorption spectra collected in this study to provide the relative proportion of the iron phases contributing to each spectrum. Reference iron *L*_2,3_-edge absorption spectra for iron in various oxidation states are provided in Fig. 4h. As differing ferric phases can provide subtly distinct *L*_2,3_-edge X-ray absorption spectra, thereby affecting fitting, a ferritin standard was used to fit the ferric component of the experimental iron *L*_2,3_-edge spectra to best represent the starting iron material.

Iron references were scaled for fitting as shown in our previous work (Everett *et al*.^58^), to enable accurate characterisation of the iron X-ray absorption spectra recorded here. The fit of the spectrum shown in Fig. 4g showed the iron content of the aggregate to be in an entirely ferric state, indistinguishable from ferritin standards (see Supplementary Fig. S3), indicating the preservation of ferritin’s ferric iron oxide core following 48 hours of incubation with Aβ.

Ferric materials provide an iron *L*_2,3_-edge absorption spectrum comprised of a dominant peak feature at 709.5 eV and a low intensity shoulder at 708 eV at the *L*_3_-absorption edge, and two further low intensity *L*_2_-absorption edge peaks at 721 and 723 eV, as shown in the reference spectrum in Fig. 4h (blue spectrum). The low-energy Fe^3+^ X-ray absorption feature at 708 eV occurs at the same energy as the principal iron *L*_3_-edge absorption feature for ferrous (Fe^2+^) and zero-oxidation-state (Fe^0^) materials. Examples of FeCl_2_ (red spectrum) and Fe^0^ (magenta spectrum) are shown in Fig. 4h. Therefore, increases in the Fe^2+^ and Fe^0^ content of an initially purely ferric iron material will manifest as an increase in the intensity of this 708 eV feature in relation to the principal *L*_3_-edge Fe^3+^ absorption peak at 709.5 eV. Likewise, at the iron *L*_2_-absorption edge, increases in Fe^2+^ and Fe^0^ content result in an increase in the intensity of the X-ray absorption feature at 721 eV with respect to the feature at 723 eV.

**Figure 5:**
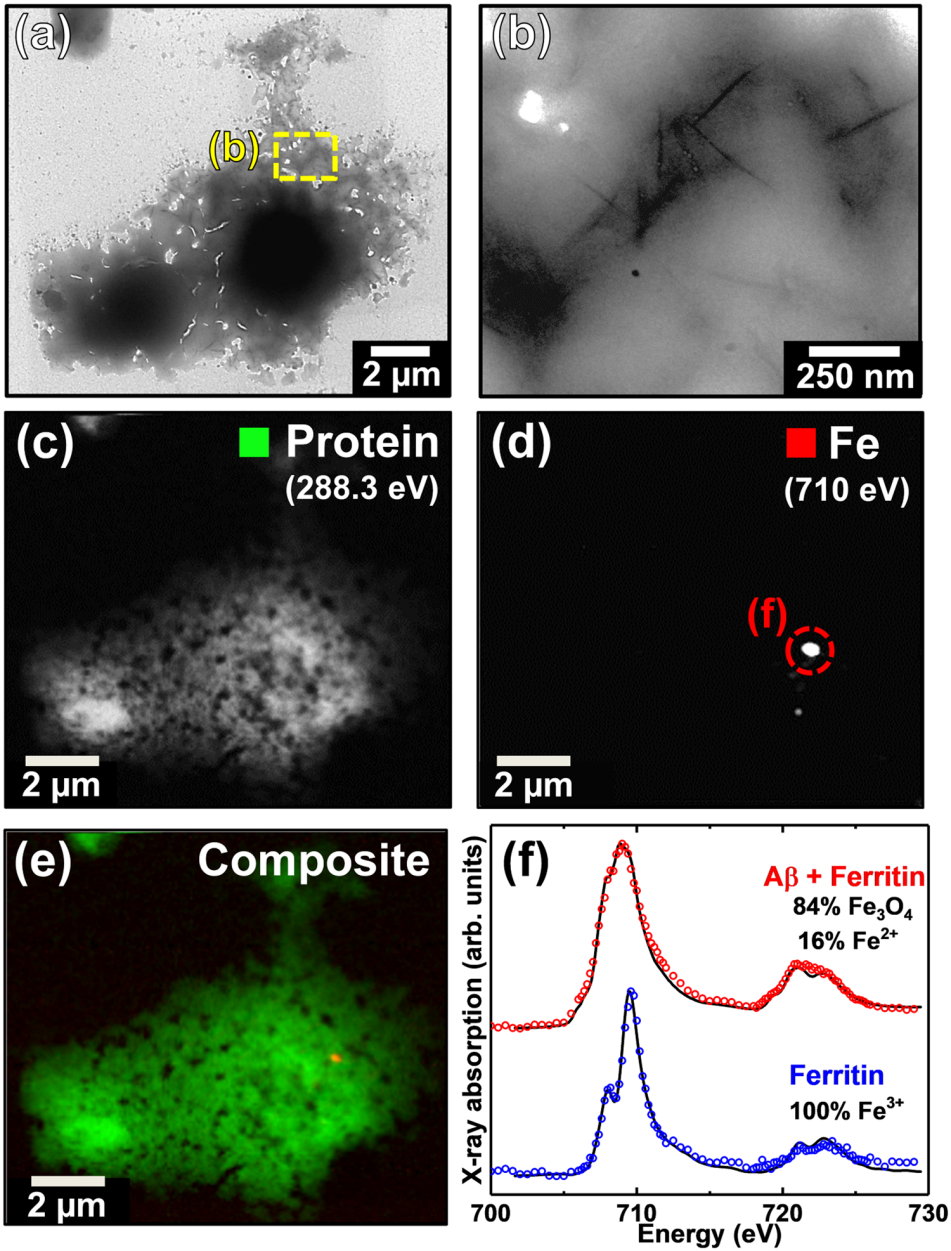
TEM and STXM analysis of an Aβ/ferritin aggregate formed following 144 hours of co-incubation. **(a,b)** TEM images of the Aβ/ferritin aggregate. STXM speciation dependent maps **(c-e)** and iron *L*_2.3_-edge X-ray absorption spectrum **(f)** from the aggregate. **(c)** Carbon *K*-edge protein map. **(d)** Iron *L*_3_-edge map. **(e)** Composite image displaying protein (green) and iron (red) content of the aggregate. **(f)** Iron *L*_2.3_-edge X-ray absorption spectra from the iron content displayed in **(d)** [red] and from a time-matched ferritin-only control [blue]. The solid lines for the spectrum corresponds to the best fit curve created by superposition of suitably scaled iron reference X-ray absorption spectra. See also Supplementary Fig. S4.

TEM and STXM images of an Aβ/ferritin aggregate formed following the full incubation period (144 hours) are displayed in Fig. 5. TEM images of this aggregate revealed a largely amorphous structure lacking any mature amyloid fibril structure (Fig. 5a,b), yet containing regions of short spiked fibrils. In contrast to the earlier time points, no electron dense fine particles were observed. STXM speciation mapping at the carbon *K*-absorption edge revealed this aggregate to be protein dense, in keeping with Aβ/ferritin structures formed following 0.5 and 48 hours of Aβ/ferritin co-incubation. However, speciation mapping at the iron *L*_3_-absorption edge showed the iron content of this aggregate to be confined to a few discrete regions (Fig. 5d), the largest of which being approximately 200 nm in diameter, rather than spatially correlated to the protein distribution of the aggregate as found in the earlier time points.

Iron *L*_2,3_-edge X-ray absorption spectra from an iron region and a time-matched ferritin control are shown in Fig. 5f. Examination of the ferritin control (Fig. 5f; blue) provided an X-ray absorption spectrum characteristic of a pure ferric phase, demonstrating that the oxidation state of iron in ferritin was maintained over the incubation period. In contrast, examination of an iron deposit within the Aβ/ferritin aggregate provided an X-ray absorption spectrum characteristic of a chemically-reduced iron phase (Fig. 5f; red). As can be seen by comparing with the Fe_3_O_4_ and Fe^3+^ standards shown in Fig. 4h (magnetite and ferritin respectively), enhanced magnetite features and diminished Fe^3+^ features were recorded across the iron *L*_2,3_-absorption edges. Fitting of the spectrum showed this area to be principally composed of magnetite (*ca*. 84%) with a minor contribution from Fe^2+^ (16%).

As no evidence of iron reduction was observed in either the ferrihydrite-like iron cores within ferritin alone, or in exposed nanoparticulate ferrihydrite time-matched controls incubated in identical buffer medium (blue trace in Fig. 5f and Supplementary Fig. S3C respectively), these alterations in iron chemistry appear to be due to the interaction of Aβ with ferritin.

Further TEM examination of sample material surrounding that shown in Fig. 5 provided evidence of a poorly fibrillar aggregate structure, similar to that shown in Fig. 4, demonstrating a heterogeneity in Aβ/ferritin aggregate morphology at this incubation point (Supplementary Fig. S4).

### Aβ/ferritin aggregates act as a site of carbonate deposition and calcium sequestration

Following our recent related observations of both calcium and carbonate within *ex vivo* human AD amyloid plaque cores using X-ray spectromicroscopy^58^, the protein, carbonate, calcium and iron contents of a further series of Aβ/ferritin incubations were investigated using the I08 beamline at Diamond Light Source, operating in the STXM mode. In these experiments, measurements were performed at the calcium *L*-edge (340–360 eV) in addition to the carbon *K*-edge and iron *L*_2,3_-edge.

**Figure 6:**
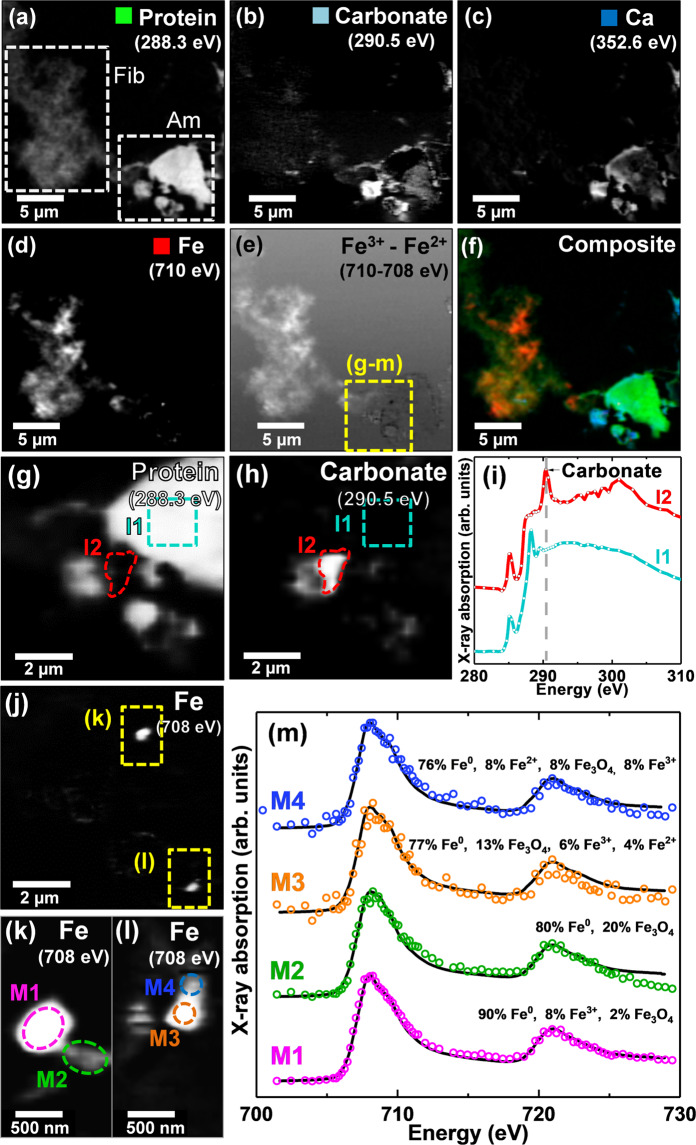
STXM analysis of an Aβ/ferritin aggregate formed following 240 hours of co-incubation. **(a)** Carbon *K*-edge protein map showing fibrillar (Fib) and amorphous (Am) protein structures. **(b)** Carbon *K*-edge carbonate map. **(c)** Calcium *L*-edge map. **(d)** Iron *L*_3_-edge map. **(e)** Iron oxidation state difference map showing regions containing predominately ferric (Fe^3+^) iron as the bright regions, and those containing predominately ferrous and/or zero-oxidation-state **(Fe**^**2+**^**/Fe**^**0**^**)** iron as the dark regions. **(f)** Composite image displaying protein (green), carbonate (light blue), calcium (blue) and iron (red) content of the aggregate. High-resolution STXM speciation **(g)** carbon *K*-edge protein map and **(h)** carbon *K*-edge carbonate map. **(i)** Carbon *K*-edge X-ray absorption spectra from the areas identified in the protein map (**g**). **(j)** High-resolution iron *L*_3_-edge map. (**k,l)**. Iron *L*_3_-edge maps of the areas highlighted in (**j**). **(m)** Iron *L*_2.3_-edge X-ray absorption spectra from the areas labelled in (**k**,**l**). The solid lines for the spectra correspond to the best fit curves created by superposition of suitably scaled iron reference X-ray absorption spectra. See Supplementary Fig. S2 Panel 2 for additional Aβ/ferritin structures from this time point.

STXM speciation maps and X-ray absorption spectra from Aβ/ferritin aggregates formed following a longer period of 240 hours of co-incubation are shown in Fig. 6. Protein speciation maps taken at 288.3 eV showed this region to contain both fibrillar-like and amorphous protein aggregates (Fig. 6a). The carbonate content of these aggregates was determined by performing speciation mapping at 290.5 eV; the energy corresponding to the absorption feature for carbonate at the carbon *K*-edge (see e.g. Figure 6i). Carbonate speciation mapping revealed that both fibrillar and amorphous aggregates contained carbonates, although the density of carbonate material was much higher in the amorphous structure (Fig. 6b).

To further investigate the origin of this carbonate material, speciation maps were performed at the calcium *L*_2_-absorption edge (352.6 eV). Calcium was found to be present in both fibrillar and amorphous aggregates with a higher density again being present in the amorphous structure (Fig. 6c). Furthermore, co-incident calcium and carbonate deposition was observed, suggesting at least some of the calcium present to be composed of calcium carbonate. However, the calcium distribution also extended beyond the carbonated areas, indicating additional calcium phases to be present.

Iron *L*_3_-absorption edge mapping (Fig. 6d) revealed the iron content of the fibrillar aggregate to be spread throughout the aggregate structure, closely following the protein morphology as was seen earlier in Fig. 4. Conversely, the iron distribution within the amorphous aggregate was found to be concentrated into dense sub-micron foci, similar to that observed in the aggregate displayed in Fig. 5, where iron was found to be in a chemically-reduced state.

To further assess the oxidation state of the iron found within these aggregate structures, an iron oxidation state contrast map was created by subtracting the STXM image taken at 708 eV (the energy of the principal Fe^2+^ and Fe^0^ iron *L*_2,3_-edge absorption peak) from the image taken at 709.5 eV (the principal Fe^3+^ peak). The resulting contrast map shows Fe^3+^ as regions of light contrast, and Fe^2+^ or Fe^0^ as areas of dark contrast (Fig. 6e). From this oxidation difference map, it is apparent that the iron associated with the fibrillar aggregate structure was in a predominantly ferric state, whereas the dense particulate iron deposits within the amorphous protein structure were predominantly ferrous or zero-oxidation-state.

To investigate this amorphous aggregate in more detail, high resolution images of the region highlighted by the yellow box in Fig. 6e were taken across the carbon *K*-edge and the iron *L*_2,3_-edge (Fig. 6g–h,j–l). The resulting carbon *K*-edge X-ray absorption spectra from region I1 (light blue trace, Fig. 6i) highlighted in Fig. 6g,h, showed the characteristic aromatic absorption peak at 285 eV and 1s-to-π* amide peak at 288.3 eV, confirming this amorphous aggregate to be composed of protein. Additionally, a carbonate absorption peak at 290.5 eV was observed in the X-ray absorption spectrum from region I2 (red trace, Fig. 6i), identified in the carbonate speciation map shown in Fig. 6h.

High resolution iron *L*_3_-edge speciation maps of this amorphous protein region revealed multiple small iron spots 200–500 nm in size (Fig. 6j–l). The iron *L*_2,3_-edge X-ray absorption spectra for these iron deposits are shown in Fig. 6m. From these spectra it appeared that the most dense iron region (labelled M1 in Fig. 6k) was composed of a strongly reduced form of iron. Fitting for this region indicated zero-oxidation-state iron (Fe^0^; see Fig. 4h, magenta for reference spectrum) to be the predominant phase (ca. 90%) with minor contributions from Fe^3+^ and Fe_3_O_4_. Although 708 eV corresponds to the principal iron *L*_3_-edge absorption feature for both Fe^2+^ and Fe^0^, the two phases are distinguishable by the broader line-shape for the Fe^0^ spectrum which lacks the splitting seen for oxide spectra, and the more prominent *L*_2_ peak and post-*L*_2_ edge absorption intensity for Fe^0^ (see Fig. 4h).

Interestingly the high resolution iron *L*_3_-edge speciation maps displayed in Fig. 6k,l showed further regions of iron deposition beyond the region identified in the lower resolution iron difference maps (Fig. 6d,e). Iron *L*_2,3_-edge absorption spectra from these further regions (labelled M2, M3 and M4) were also consistent with a heavily reduced iron phase. Fitting of these spectra showed all of these regions to be primarily composed of Fe^0^, with minor contributions from Fe_3_O_4_, Fe^3+^ and Fe^2+^. This confirmed that low-oxidation-state iron material was diffusely spread throughout the aggregate, as opposed to punctate sources which could conceivably be artefacts arising from external nanoparticulate sources. Further examples of Aβ/ferritin aggregates formed from this Aβ/ferritin series following 240 hours of incubation are displayed in the Supplementary Fig. S2 (Panel 2). As was the case in Fig. 6, diffuse iron associated with these aggregates was found to be entirely ferric, whereas dense sub-micron iron deposits were found to be chemically reduced.

## Discussion

From the data presented here, it is apparent that Aβ(1-42) interacts with ferritin in a manner that leads to the conversion of ferritin-encapsulated ferric iron into nanoscale deposits of chemically reduced iron. These findings implicate an Aβ/ferritin interaction in the formation of the nanoscale ferrous-rich and zero-oxidation-state iron minerals previously observed in tissue from AD cases^48–50^, transgenic AD mouse tissues^57^, and isolated AD pathological structures^47,58^. In addition, Aβ/ferritin aggregates were shown to sequester calcium and act as a site of carbonate deposition, resembling such mineralised deposits found in AD amyloid plaque core material^58^.

Electron microscopy, time-lapse imaging and X-ray spectromicroscopy (STXM) demonstrated that co-incubation of Aβ(1-42) with ferritin, results in the accumulation of ferritin within Aβ-aggregate structures. Ferritin was found to be widely spread throughout amyloid aggregates over the first 48 hours of co-incubation, with X-ray spectromicroscopy speciation mapping showing that the iron content closely follows peptide morphology, a finding in keeping with our previous *in vitro* examination of Aβ/ferrihydrite interaction (see Everett *et al*.^59^). The inclusion of ferritin within amyloid aggregates resulted in poorly defined peptide structures when compared to Aβ incubated in isolation. This dependence of Aβ fibril morphology on the presence/absence of ferritin may arise through interactions between the two species (potentially influencing the formation of secondary and tertiary Aβ structures), and/or through localized variations in peptide concentration arising from the rapid co-aggregation of Aβ and ferritin as shown in Fig. 1. The ability of Aβ to accumulate and incorporate ferritin into its structure as found here, also provides an explanation for observations made by Grundke-Iqbal *et al*. where ferritin was observed to be co-localised with senile plaque material in AD tissues^55^.

Prolonged incubation of Aβ(1-42) with ferritin under sterile conditions over longer time periods of 144–240 hours led to the observation of both poorly defined fibril structures (as observed over the first 48 hours of incubation) and amorphous aggregate structures. TEM imaging of these amorphous structures showed a lack of amyloid fibril morphology, coupled with an absence of the electron-dense particulate material that would indicate intact ferritin. This is consistent with either disruption of the ferritin structure and/or the removal of the iron oxide core from the ferritin cage (the ferritin iron oxide core provides the strong TEM contrast in unstained images). As mature amyloid fibril structures were observed when Aβ was incubated in the absence of ferritin, and electron-dense cores indicating intact ferritin were maintained when ferritin was incubated in isolation, these alterations to amyloid structure appear to be a direct result of the Aβ-ferritin interaction.

STXM speciation mapping showed that the iron distribution within aggregate structures formed following 144–240 hours of incubation varied dramatically, and depended on amyloid morphology. Where aggregates were composed of poorly defined fibrils, iron was diffusely spread throughout the aggregate, closely following protein distribution. Conversely, where aggregates were present in an entirely amorphous state, iron was no-longer diffusely spread throughout the aggregate, instead being localised into dense foci. Examination of aggregate structures across the entire iron *L*_2,3_-absorption edge demonstrated the oxidation state of ferritin-derived iron to also be dependent upon both amyloid and iron morphology. Where the amyloid and iron content was “fibrillar” in nature, iron was found to remain in a ferric state. However, in amorphous amyloid aggregates where iron was localised in dense deposits hundreds of nanometers in diameter, iron was in a chemically-reduced state. Thus, through the use of STXM we provide the first descriptions of intra-sample variations in amyloid morphology dependent upon iron oxidation state, heterogeneity which is undetectable with standard bulk sample measurements of fibril formation.

This association between amyloid aggregate morphology and iron oxidation state was reproducibly observed across multiple, independent, Aβ/ferritin incubation series, examined at different X-ray spectromicroscopy beamlines, and using different starting batches of Aβ. Fitting of the iron *L*_2,3_-edge absorption spectra from these chemically-reduced iron regions showed the presence of a magnetite-like ferrous-rich material and an additional further-reduced iron phase with absorption features consistent with zero-oxidation-state iron. These Fe^0^ phases are analogous to those we reported in our *ex vivo* examination of amyloid plaque material extracted from AD grey matter, suggesting a similar phase to be present^58^. As no evidence of reduced iron was observed where ferritin or ferrihydrite was incubated in the absence of Aβ, the creation of a reducing environment and changes in iron chemistry appear to be driven by the co-aggregation of Aβ and ferritin. The absence of detectable low-oxidation-state iron in the ferritin controls also demonstrates that the chemically-reduced iron observed within the Aβ/ferritin aggregates is unlikely to be from iron bound to the external surface of ferritin, where surface iron can arise as an artefact of ferritin purification.

The identification of nanoscale deposits of chemically-reduced iron further demonstrates the necessity for chemically-sensitive nanoscale resolution microscopy when examining the chemistry of Aβ/iron interactions. These deposits would not have been detected using bulk measurements or microfocus microscopy, where the signal from the reduced iron phases would have been lost in the prevailing signal arising from oxidized iron.

The ability of Aβ to influence the chemical composition of ferritin’s ferrihydrite core, resulting in the formation of a chemically-reduced iron phase, is entirely consistent with our previous X-ray based *in vitro* experiments where Aβ(1-42) was shown to induce the chemical reduction of ferric oxyhydroxide and ferrihydrite into a pure ferrous phase^59,60^. Our previous experiments were conducted using iron oxide phases directly exposed to Aβ. It was not known if ferritin-encapsulated ferric iron oxide cores could be affected, although our original study of iron oxide nanoparticles in extracted amyloid plaque cores pointed to ferritin as a potential source of the iron due to the size distribution of the measured particles^47^. In the present work, for the first time, Aβ(1-42) was shown to indeed be capable of influencing the redox chemistry of iron originating within the ferritin protein. Our previous and present findings parallel a recent *in vitro* study by Balejcikova *et al*. who utilised spectrophotometry to record a moderate increase in Fe^2+^ iron content when a different peptide fragment, Aβ(1-40) was incubated with ferritin, compared to where ferritin was incubated in isolation^61^.

What remains unclear is whether these changes to ferritin iron oxidation state occur whilst iron remains within the ferritin cage (i.e. electron transfer across the apoferritin protein as described in Watt *et al*.)^74^, or whether iron must first be extracted from the ferritin cage before reduction can take place. In either scenario, these changes would appear to be dependent on Aβ amyloid interaction with ferritin. Time-matched control experiments conducted in the absence of Aβ showed no evidence of iron leaching from the ferritin-cage, or of chemically-reduced iron (i.e. only ferric iron was detected).

*In vitro* evidence shows the reduction of ferritin-encapsulated iron to a ferrous state to be an effective method for the removal of iron from the ferritin cage^16^. If the Aβ/ferritin interaction creates an environment in which iron is chemically-reduced via electron transfer across the ferritin cage, iron may be exuded from ferritin, leading to an increased labile iron pool. Iron located in channels near the surface of the ferritin protein cage, critically involved in the transfer of iron between transferrin and ferritin, may represent a physiologically relevant iron source, susceptible to Aβ-mediated reduction^75,76^. Indeed, this iron has been shown to be rapidly extracted from ferritin by the iron chelator desferrioxamine^75,76^. This highlights the clinical importance of determining if ferritin protein quantified as a measure of iron status has retained its iron or whether it is effectively apoferritin^77^. In this context, quantifying apoferritin:ferritin levels (for example in the CSF) might have potential as a marker for amyloid/ferritin interaction *in vivo*. Additionally, the low level of free-iron we recorded in the starting ferritin suspensions may have acted for a seed for Aβ-mediated iron reduction, in a manner similar to our previous studies^59,60^. This scenario is certainly possible *in vivo*, should the ferritin protein cage be compromised, thereby exposing the ferritin iron core.

Under normal conditions *in vivo*, where iron homeostasis is well-managed, intracellular ferritin expression depends on levels of intracellular labile iron. If Aβ has the capacity to disrupt ferritin storage resulting in the chemical reduction and release of iron, it is conceivable that this could compromise intracellular iron metabolism to the extent that antioxidant defences are overwhelmed. The observation of chemically-reduced iron as dense iron foci is also consistent with the iron nucleation processes observed during the chemical synthesis of mixed valence^78^ and zero-oxidation-state iron nanoparticulates from unbound ferric iron precursors^79^.

Whilst these findings clearly show Aβ interaction with ferritin results in the chemical reduction of ferritin-derived iron, the reductant itself is still to be confirmed. Aβ may act as a reducing agent for iron in a manner akin to that originally proposed by Huang *et al*.^10^, with this possibility currently under further investigation. Alternatively, the co-aggregation of Aβ with ferritin could result in the formation of highly-localised compartments in which reactants capable of influencing ferritin-iron chemistry can accumulate. A possible example for this scenario is the blocking of ferritin’s hydrophilic channels by Aβ, preventing the escape of reactants such as superoxide radicals (formed at the ferritin ferroxidase centres), resulting in the chemical reduction of ferritin-iron. Indeed, the chemical reduction and subsequent release of ferritin iron via a superoxide-dependent mechanism has been demonstrated *in vitro*^80,81^.

These results suggest that not only labile iron pools but also ferritin-encapsulated iron may act as a source of chemically-reduced forms of iron in AD tissue. This may account for the increased levels of low-oxidation-state iron derived from ferritin isolated from AD tissues^50^, the ferritin-core sized magnetite-like deposits previously identified within amyloid plaque material using HR-TEM^47^, and the magnetite particles of proposed biogenic origin recorded in human brain tissues through isothermal resonance magnetisation experiments^82^. This mechanism is far from implausible, as shown through the artificial production of chemically-reduced iron nanoparticles within the apoferritin cage, by first removing the natural ferritin core by iron reduction, with the subsequent use of iron Fenton chemistry to biomineralise the new magnetite nanocrystals^83^.

Ferritin is abundant throughout the human brain^1,12^, and has been observed to accumulate in localised regions of Aβ deposition^55,84^, whilst microglia, known to secrete the ferritin protein^85^, have also been associated with senile plaques in AD tissues^55^ providing potential routes for ferritin to interact with Aβ structures *in vivo*. Further evidence of ferritin accumulation in regions of Aβ pathology has recently been shown in 5XFAD transgenic mouse hippocampal tissue, where antibodies specific for ferritin were found to accumulate in brain regions with both increased ferritin expression and a high loading of Aβ plaques^86^.

Interactions between aggregating Aβ and ferritin may represent significant sources of localised oxidative stress in AD tissue, potentially contributing to the neurodegeneration associated with AD^8,9,62–64^. It is noteworthy that others have previously observed associations between ferritin and aberrant tau filament structures as observed in AD and progressive supranuclear palsy patients, and that disruption to the function of ferritin can be detrimental to the many iron-dependent functions in the brain, in extreme cases (such as neuroferritinopathy) proving fatal^84,87–92^.

The association of largely amorphous amyloid structures with the occurrence of chemically-reduced iron deposits several hundred nanometers in diameter as observed here, was also reported in our X-ray spectromicroscopy examination of cortical tissue from APP/PS1 mice^57^, and isolated amyloid plaque material from AD subjects^58^; suggesting that Aβ/ferritin interaction may have acted as source for the formation of chemically-reduced iron in these instances. The identification of differing amyloid/ferritin aggregate subtypes ([i] fibrillar amyloid aggregates containing diffuse ferric iron, [ii] amorphous amyloid aggregates containing dense chemically-reduced iron), indicates that the chemical state of amyloid-associated iron may reflect the type of amyloid aggregate that has formed (Table 1).

**Table 1:** Summary of the differing aggregate types observed in this study. Identified iron phases and the presence of calcium and carbonate loading are shown for the cases where such measurements were obtained. “—” denotes not measured.

Sample Type	Incubation Time (hrs)	Aβ morphology	Iron Distribution	Iron oxidation state	Calcium Loading	Carbonate Loading
Aβ(1-42)	All time points	Fibrillar	N/A	N/A	—	None
Ferritin	All time points	N/A	N/A	Ferric (Fe^3+^)	—	None
Aβ(1-42) +Ferritin	0.5	Fibrillar	Uniform throughout aggregate	Ferric (Fe^3+^)	—	—
	48	Fibrillar	Uniform throughout aggregate	Ferric (Fe^3+^)	—	—
	144	Fibrillar	Uniform throughout aggregate	—	—	—
		Amorphous	Localised deposits (sub micron)	Mixed Valence (Fe^2+^/Fe^3+^)	—	—
	240	Fibrillar	Uniform throughout aggregate	Ferric (Fe^3+^)	Low	Low
		Amorphous	Localised deposits (sub micron)	Mixed Valence (Fe^2+^/Fe^3+^)/Zero Valence (Fe^0^)	High	High

In addition to alterations in native iron chemistry, STXM examination of Aβ/ferritin aggregates formed following 144–240 hours of interaction at the carbon *K*-edge revealed the presence of additional carbon containing species. Regions of carbonate were located in Aβ/ferritin structures demonstrating carbonate to have been deposited during the co-aggregation of Aβ and ferritin, with protein N-terminal carbonates or dissolved CO_2_ as a potential source. As calcium was present in the buffer, examination of the amorphous Aβ/aggregate structure formed following 240 hours of incubation (shown to contain low-oxidation-state iron) was also performed at the calcium *L*-edge. Calcium *L*-edge speciation mapping revealed co-localised carbonate and calcium deposition, suggesting carbonate material to be comprised of calcium carbonate. Calcium carbonate formation therefore appears to be linked to Aβ/ferritin interaction.

Remarkably, this pattern of calcium mineralisation, carbonate loading, and nanoscale regions of low-oxidation-state iron formation within Aβ/ferritin aggregates structures is entirely consistent with that observed in amyloid plaque core material extracted from individuals with confirmed AD, as recently reported by our group^58^. These striking similarities are displayed in the comparison Fig. S5 of the Supplementary Information. This suggests that amyloid plaques may be formed through a similar process of Aβ/ferritin interaction in the presence of calcium *in vivo*. In particular, the characteristic features of dense amorphous peptide aggregates containing deposits of Fe^2+^, magnetite or Fe^0^, were replicated in the *ex-vivo* plaque core material.

In conclusion, the data presented here demonstrate that the interaction of Aβ with ferritin in a physiologically relevant buffer medium results in the formation of dense amorphous amyloid structures harbouring chemically-reduced iron. This points towards Aβ/ferritin interaction as a probable source of the increased levels of mixed oxidation state Fe^2+^ and Fe^0^ phases previously observed within brain tissue from the APP/PS1 model of AD, in human AD tissue, and in isolated amyloid plaque cores^47–49,57,58,93^. Furthermore, the chemical composition of the *in vitro* aggregates formed in this study was shown to be analogous to primary components of the amyloid plaque core material extracted from the grey matter of AD subjects^58^, consistent with Aβ/ferritin interaction playing a prominent role in the development of iron-bearing amyloid deposits *in vivo*.

Given the abundance of ferritin throughout the human brain^1^, the capacity for Aβ to create an environment in which ferritin iron is chemically reduced, could lead to the sustained production of excess ROS, and therefore to oxidative stress in AD tissues. With oxidative stress being a key characteristic of AD pathology^62,63,65,94^, this interaction could represent a target for therapies intended to lower oxidative burdens and delay disease progression, either through targeting Aβ-induced low-oxidation-state iron phases, or by preventing Aβ from interacting with ferritin and so averting the formation of chemically-reduced iron. Moreover, as specific iron and calcium phases provide marked and quantifiable impact on MRI contrast, it is possible that forms of iron and calcium preferentially associated with amyloid deposits could be used as endogenous markers to facilitate screening for amyloid deposition in at-risk populations, before clinical manifestations arise.

## Methods

### The Interaction of Aβ and Ferritin

#### Preparation of Aβ/ferritin Suspensions

Horse spleen ferritin (Type I; 125 mg/mL; 1% saline solution) was purchased from Sigma Aldrich and stored at 4 °C until time of use. Characterisation of ferritin size, crystal structure, oxidation state and iron content are provided in the Supplementary Information (Figs. S1, S3 and S6). Ferritin was diluted in a modified Krebs-Henseleit (KH) buffer (pH 7.4; 100 mM PIPES (piperazine-N,N′-bis(2-ethanesulfonic acid), 118.5 mM NaCl, 4.8 mM KCl, 1.2 mM MgSO_4_, 1.4 mM CaCl_2_, 11 mM glucose) to achieve a 0.7 mg/mL (440 µM iron) ferritin suspension. KH buffer was made on the day of use, and was filtered (0.2 µm pore size) in sterile conditions prior to use, to inhibit bacterial growth. This buffer medium, modelled on the cerebral spinal fluid which bathes the brain, contains physiologically relevant concentrations of Ca^2+^ and Mg^2+^, both of which have been shown to influence amyloid peptide aggregation dynamics^95,96^. The buffer PIPES was chosen as it does not strongly interact with metal ions^97^.

Synthetic monomeric Aβ(1-42) (Bachem) was thawed and dissolved in 1 mM NaOH (Sigma Aldrich) to create a 1 mg/mL (220 μM) stock. Aβ stock was allowed to sit at room temperature for 30 minutes to ensure complete peptide dissolution before being added to 0.7 mg/mL ferritin suspensions in KH buffer under sterile conditions. Final ferritin and Aβ concentrations were 0.6 mg/mL (1.3 µM, 370 μM iron content) and 0.16 mg/mL (35 μM) respectively. A ferritin concentration of 0.6 mg/mL was used as this value corresponded to the iron concentration used in our previous *in vitro* experiments examining Aβ interaction with ferric iron^59,60^. This ferritin concentration ensures sufficient iron is present to enable detection of iron using X-ray spectromicroscopy when employing the sampling technique described below. Aβ-free ferritin controls were created as above, with the substitution of 1 mM NaOH for Aβ. 35 μM Aβ solutions were prepared in KH buffer as an amyloid reference. 370 µM 6-line ferrihydrite suspensions were prepared in KH buffer as an unencapsulated iron reference. Ferrihydrite was synthesised as described in Schwertmann *et al*.^98^.

#### Incubation of suspensions

All suspensions/solutions were incubated under aerobic conditions at 37 °C within sealed microcentrifuge tubes until the time of sampling. For time lapse imaging a camera was mounted inside the incubator, allowing images of Aβ/ferritin suspensions to be taken *in situ* without physical manipulation of the sample tubes. Time lapse images demonstrating the precipitation of ferritin by Aβ were created using Lapse it™ time lapse software. Images of microcentrifuge tubes containing Aβ/ferritin and a ferritin only control were taken over a period of 18 hours.

The incubation of ferritin suspensions in KH buffer did not result in the leaching of ferritin iron, as demonstrated through iron quantification measurements performed over a 240 hour incubation period (see Supplementary Information).

#### Scanning Transmission X-ray Microscopy Sample Preparation

15 μL of Aβ/ferritin suspensions were deposited onto either silicon oxide membranes (DuneSciences; 75 nm thickness) or silicon nitride membranes (Agar Scientific; 75 nm thickness) and excess liquid was removed using filter paper. For the Aβ/ferritin series examined on the PolLux beamline at the Swiss Light Source sampling was performed following 0.5, 48 and 144 hours of incubation. An additional Aβ/ferritin series was examined using the I08 beamline at Diamond Light Source, with sampling being performed following 240 hours of incubation. Additional Aβ and ferritin control samples were examined to provide reference carbon *K*-edge and iron *L*-edge reference X-ray absorption spectra.

As STXM was the primary technique employed to assess iron oxidation state following Aβ/ferritin incubation, conscientious effort was taken to maintain anoxic conditions during sampling, transfer and examination of Aβ and ferritin materials. All STXM samples were prepared within a nitrogen filled glove bag (Glas-col), and membranes containing Aβ/ferritin structures were stored within a nitrogen filled O-ring sealed jar (Oxoid) to prevent changes in iron oxidation states. Membranes were mounted onto STXM sample holders for X-ray spectromicroscopy. Sample mounting was conducted within a nitrogen filled glove bag. Anoxic conditions were maintained during sample transportation by using a nitrogen filled vessel for sample transfer and purging of the STXM endstation with nitrogen prior to sample loading.

#### Electron microscopy sample preparation

Small volumes of Aβ/ferritin suspensions were deposited onto carbon/formvar coated copper TEM grids and excess liquid removed. Samples were taken following 0.5, 48, 96 and 144 hours of incubation.

#### X-ray spectromicroscopy: scanning transmission X-ray microscopy

X-ray spectromicroscopy was performed at the Swiss Light Source on the PolLux beamline using the STXM endstation, and at the Diamond Light Source on beamline I08. Focussed X-ray spot size was approximately 20 nm on the PolLux beamline and 50 nm on I08. Energy-specific images of sample material were created by raster scanning the sample across a focussed X-ray beam and recording the intensity of the transmitted X-rays. To minimise potential photoreduction effects caused by the incident X-ray beam thereby preserving native sample chemistry, scanning (exposure) times were kept to a minimum (typically 1–2 ms per point).

To create contrast maps displaying the chemical speciation of a given sample material, paired images were taken at the energy of a feature of interest (e.g. the main ferric (Fe^3+^) iron peak in the Fe *L*_3_-edge absorption region) and a few eV below this feature. The “off-peak” images were then subtracted from the “on-peak” images revealing the chemical speciation for the examined sample region.

X-ray absorption spectra were collected by taking a series of images (called a stack) of a region of interest at multiple energies across a desired energy range (e.g. the iron *L*_2,3_-absorption edge [700–740 eV], or the carbon *K*-absorption edge [280–310 eV]). Transmitted X-ray intensities for the stack images were converted to optical density using background regions that did not contain any sample material, thereby removing background X-ray absorption features attributable to the beamline. This method of spectromicroscopy allows an X-ray absorption spectrum to be created from every pixel of a stack image, thus allowing spectral information to be realised from highly localised regions of interest.

Carbon *K*-edge X-ray spectromicroscopy was performed prior to higher energy iron *L*_*2,3*_-edge examination as to minimize X-ray beam induced damage to carbon structures. Only a sub-set of Aβ/ferritin structures were examined using STXM due to experimental time constraints.

X-ray spectromicroscopy data were processed using the aXis 2000 software package (http://unicorn.mcmaster.ca/aXis2000. html). The brightness and contrast of X-ray microscopy images were adjusted using ImageJ software. Grey scale X-ray microscopy images were converted to false colour before being recombined as overlays to create coloured composite images.

### Transmission electron microscopy

Transmission electron microscopy (TEM) was performed using a JEOL 1230 microscope operating at 100 kV. A low operating energy was used to minimise electron beam damage of sample materials. No positive or negative stains were used during TEM microscopy. Where both techniques were employed, TEM was performed following STXM examination to prevent electron beam induced changes to sample chemistry.

### Quantification and statistical analysis

#### Analysis of X-ray Absorption Spectra

To estimate the relative proportion of iron phases contributing to the iron *L*_2,3_-edge X-ray absorption spectra measured in these experiments, iron *L*_2,3_-edge X-ray absorption spectra were fitted to reference X-ray absorption spectra from Fe^3+^, Fe^2+^, Fe_3_O_4_ and Fe^0^ standards using non-linear least squares fitting procedures. Accurate scaling of these standards was required to provide precise quantitative determination of the phase proportions contributing to the experimental data. The scaling factors were determined by normalising the X-ray absorption intensity for each reference iron phase to the integrated intensity over the iron *L*_2,3_ absorption edges, as described in our previous work (see Everett *et al*.^58^). An iron *L*_2,3_-edge X-ray absorption spectrum from a ferritin standard was used for fitting the Fe^3+^ contribution to the experimental iron *L*_2,3_-edge X-ray absorption spectra. The absorption features from this ferritin spectrum are consistent with a pure ferric phase, and it was therefore chosen as a suitable ferric (Fe^3+^) standard for fitting. The Fe^0^ reference spectrum used for fitting was obtained from Fe^0^ film standards prepared and measured under vacuum to prevent oxidation^99^.

## Supplementary information

Supplementary Information.

## Data Availability

The datasets generated during and/or analysed during the current study are available through the Keele Research Repository at https://eprints.keele.ac.uk.
