# Supplementary material for: Iron stored in ferritin is chemically reduced in the presence of aggregating Aβ(1-42)
Source: Sci Rep. 2020 Jun 25;10:10332. doi: 10.1038/s41598-020-67117-z (PMC7316746; doi:10.1038/s41598-020-67117-z)
Supplement: Supplementary file 1 — Supplementary Information. [file 41598_2020_67117_MOESM1_ESM.pdf]

Supplementary Information: Iron stored in ferritin is chemically reduced in the presence of aggregating Aβ(1-42)

James Everett[1,2*], Jake Brooks[2], Frederik Lermyte[2,3], Peter B. O'Connor[3], Peter J. Sadler[3], Jon Dobson[4,5], Joanna F. Collingwood[2,5] and Neil D. Telling[1]

[1]School of Pharmacy and Bioengineering, Keele University, Stoke-on-Trent, Staffordshire, ST4 7QB, United Kingdom

[2]School of Engineering, University of Warwick, Coventry, CV4 7AL, United Kingdom

[3]Department of Chemistry, University of Warwick, Coventry, CV4 7AL, United Kingdom

[4] J. Crayton Pruitt Family Department of Biomedical Engineering & Department of Materials Science and Engineering, University of Florida, Gainesville, Florida 32611, United States

[5]Department of Materials Science and Engineering, University of Florida, Gainesville, Florida 32611, United States

*Corresponding author: j.everett@keele.ac.uk

Table S1. Aβ(1-42) batches and X-ray spectromicroscopy beamlines used to generate the data shown in Figures 1 – 6 and S1-S5.

**Aβ(1-42) Batch**	**Investigation**	**Figure(s)**
1	Time dependent visual observation of Aβ(1-42) aggregation	1
2	TEM	2, S1
3	STXM: PolLux beamline	3
4	STXM: PolLux beamline/TEM	4, 5, S2 (Panel 1),S4
5	STXM: I08 beamline	6, S2 (Panel 2), S5

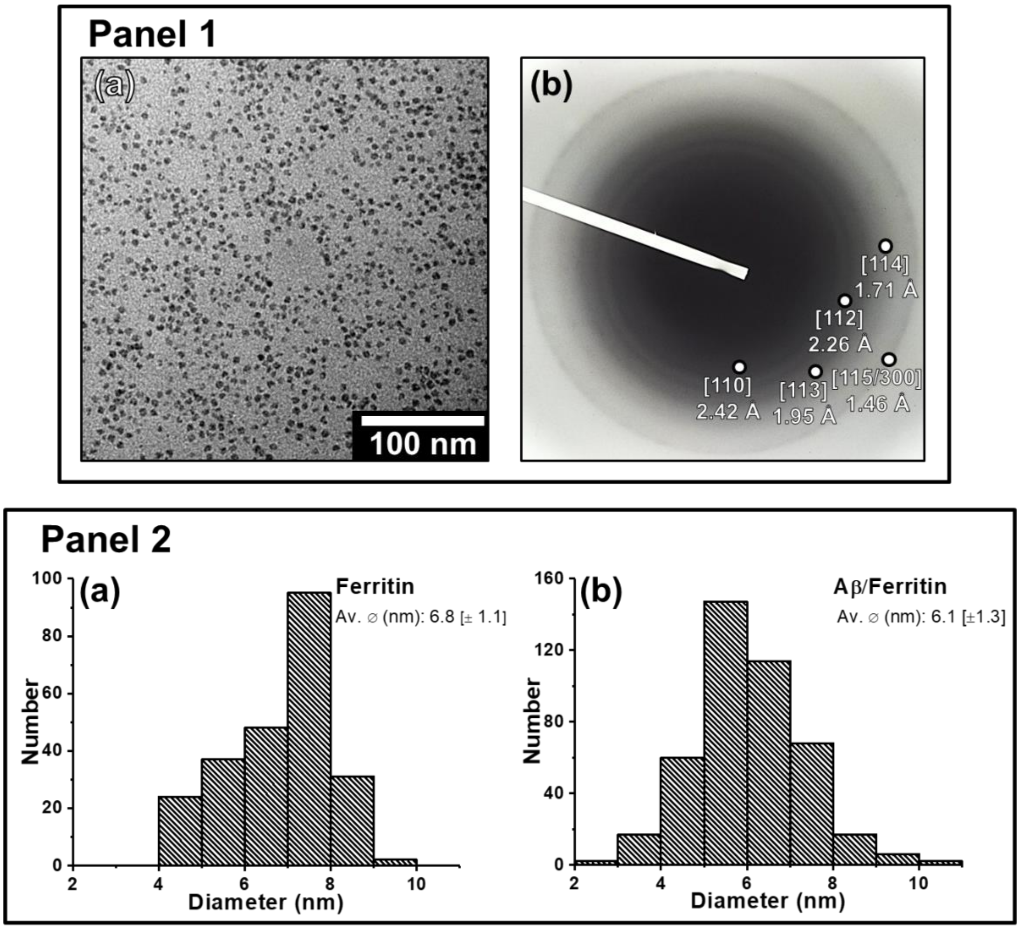

Figure S1. Characterisation of ferritin particles. **Panel 1 – (a)** TEM images and **(b)** electron diffraction patterns from an unstained uniform ferritin sample. Electron diffraction provided ringed pattern and *d*-spacing values consistent with 6-line ferrihydrite as shown in Table S2. **Panel 2 -** Size distribution of electron dense particulates recorded in TEM images shown in Panel 1 and Figure 2. Average diameter size was **(a)** 6.8 nm [±1.1] for Ferritin only (n = 237) and **(b)** 6.1 nm [±1.3] for Aβ/Ferritin (n = 434) sample material.

Table S2. Ferritin *d*-spacing values. *d*-spacing values obtained from the ringed patterns shown in Figure S1 compared to a ferrihydrite standard (Drits *et al.* [1]).

***d*-spacing (nm) {relative intensity}**	
**Ferritin (standard error)**	**Ferrihydrite (hkl)**
0.242 (±0.005){100}	0.25 (110){100}
0.226 (±0.008){100}	0.224 (112){80}
0.195 (±0.08){30}	0.197 (113){35}
0.171 (±0.07){30}	0.172 (114){30}
-	0.151 (115){50}
0.146 (±0.05){70}	0.147 (300){70}

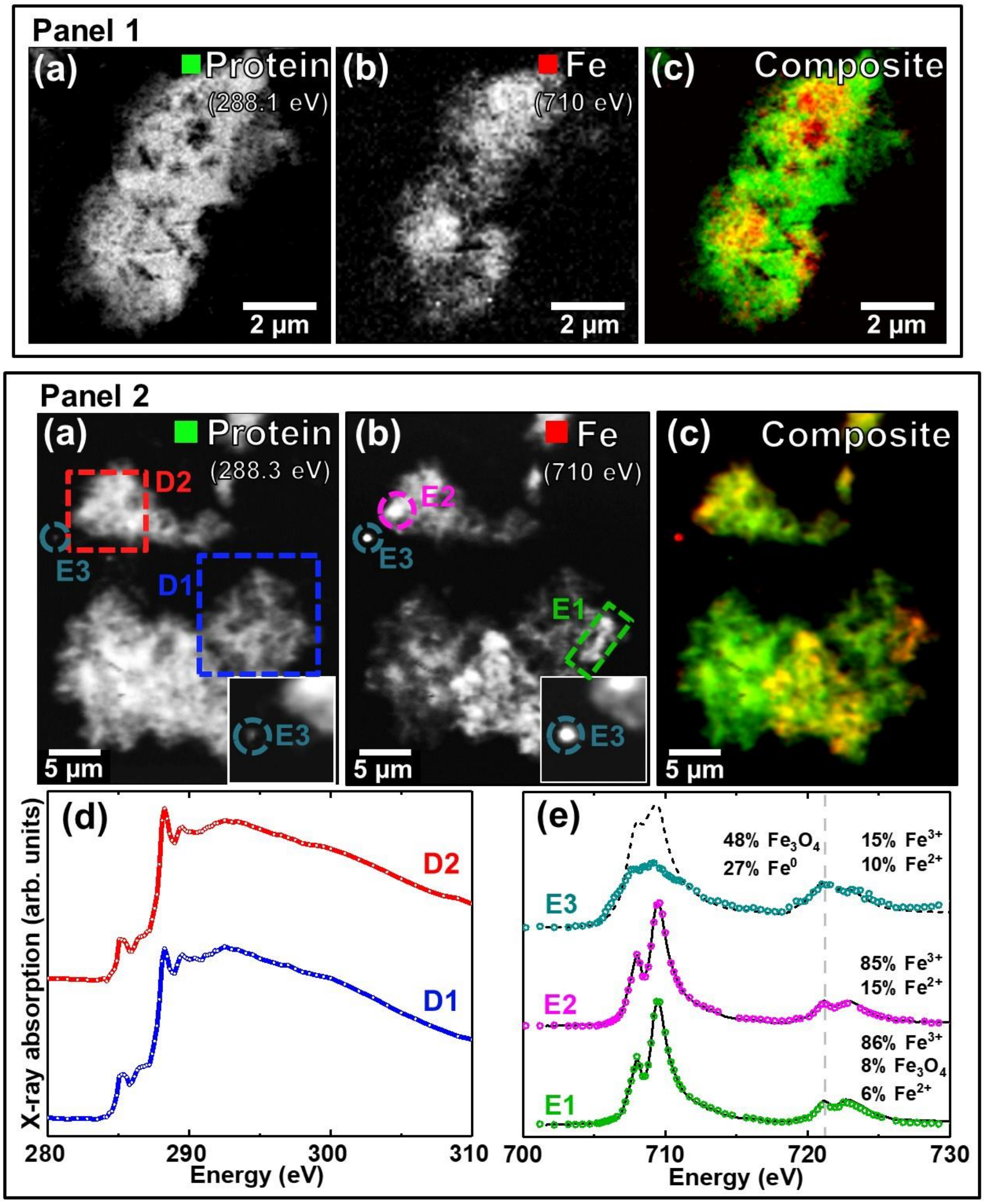

Figure S2. STXM examination of additional Aβ/ferritin aggregates. **Panel 1 -** STXM speciation dependent maps from an Aβ/ferritin aggregate formed following 0.5 hours of co-incubation. **(a)** Carbon *K*-edge protein map. **(b)** Iron *L*-edge map. **(c)** Composite image displaying protein (green) and iron (red) content of the aggregate. **Panel 2 -** STXM speciation dependent contrast maps, carbon *K*-edge X-ray absorption spectra and iron $L_{2.3}$-edge x-ray absorption spectra from Aβ/ferritin aggregates formed following 240 hours of co-incubation. **(a)** Carbon *K*-edge protein map. **(b)** Iron *L*-edge map. **(c)** Composite image displaying protein (green) and iron (red) content of the aggregate. **(d)** Carbon *K*-edge X-ray absorption spectra from the areas identified in the protein map (a). **(e)** Iron $L_{2,3}$ X-ray absorption spectra from the

areas highlighted in the iron map (b). Examination of iron associated with fibrillar carbon morphology in this aggregate provided iron $L_{2,3}$-edge X-ray absorption spectra characteristic of a ferric material. Conversely iron $L_{2,3}$-edge examination of a dense iron deposit (ca. 500 nm diameter) associated with carbon, located approximately 1 µm away from the main aggregate body (highlighted as area E3 in Fig. S2, Panel 2b), provided an X-ray absorption spectrum consistent with a chemically reduced iron phase. Despite clear saturation effects at the L_3 edge, manifesting in the apparent enhancement of the L_2 to L_3 peak ratio, this iron was determined to be chemically-reduced due to the enhanced Fe^{2+} features and diminished Fe^{3+} features recorded at the low-intensity iron L_2-absorption edge where saturation effects do not contribute to the X-ray spectrum. Fitting of this spectrum over the **L_2-edge only**, indicated this area to be comprised of primarily magnetite and Fe^0 with minor contributions from both Fe^{3+} and Fe^{2+} cations. This dense particulate iron deposit was similar in morphology and size to that shown in Fig. 5, which was also found to be in a chemically reduced state (Fig. 5f). Best fit curves were created by superposition of suitably scaled iron reference X-ray absorption spectra as described in Everett *et al.* 2018 [2]. Of the 10 Aβ/ferritin aggregates examined over the entire iron $L_{2,3}$-edge using STXM, 5 were found to contain chemically-reduced iron (50%). Within this population, 1-2 reduced iron particulates were identified per aggregate.

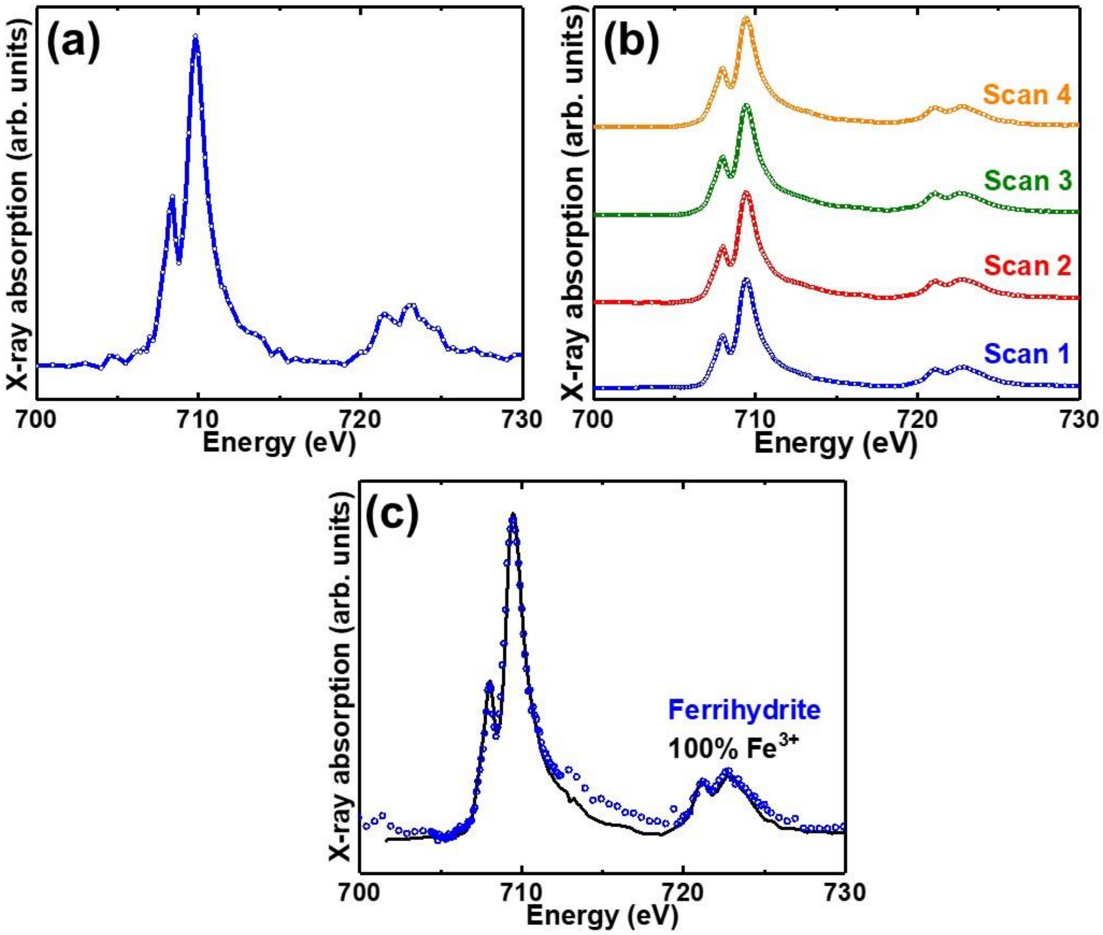

Figure S3. Ferritin and ferrihydrite x-ray absorption spectra. **(a)** Iron $L_{2,3}$-edge absorption spectrum from ferritin acquired at Diamond Light Source beamline I08. This spectrum is characteristic of a pure ferric (Fe^{3+}) phase. **(b)** Iron $L_{2,3}$-edge absorption spectra from ferritin collected over four successive scans performed at Diamond Light Source beamline I10. Sample preparation and examination for (b) followed the methodology described in Everett *et al.* 2014 [3,4]. **(c)** Ferrihydrite iron $L_{2,3}$-edge absorption spectrum (blue) collected at Diamond Light Source beamline I08. Ferrihydrite was incubated in KH buffer for 240 hours at 37 ˚C. The best fit curve for this spectrum (black line) was consistent with a pure ferric material, indicating no chemical reduction of ferrihydrite occurred during incubation in the buffer medium. Best fit curves were created by superposition of suitably scaled iron reference X-ray absorption spectra as described in Everett *et al.* 2018 [2].

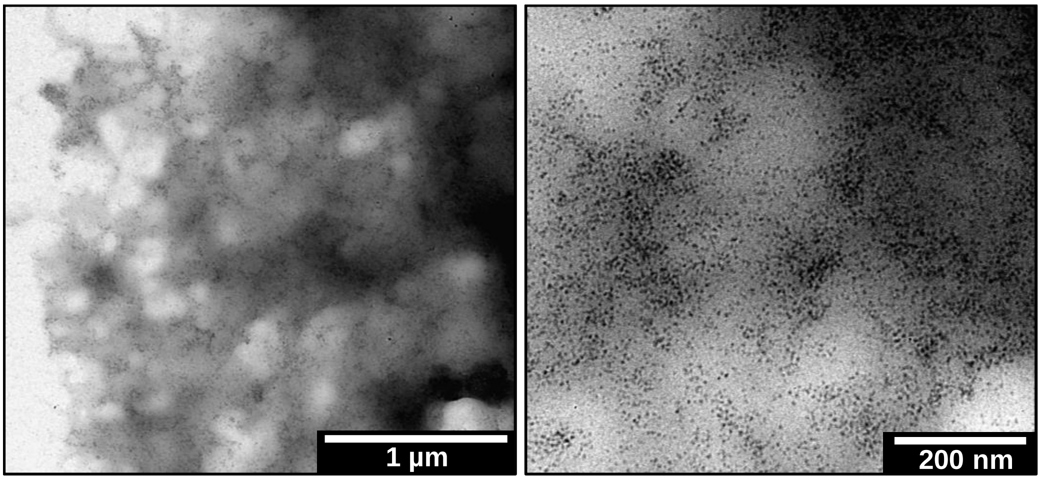

Figure S4. TEM images of an additional Aβ/ferritin aggregate. TEM images of an Aβ/ferritin aggregate found in close proximity to the aggregate structure shown in Fig. 5 of the main text. The aggregate is poorly fibrillar in structure containing a large number of electron dense ferritin particles.

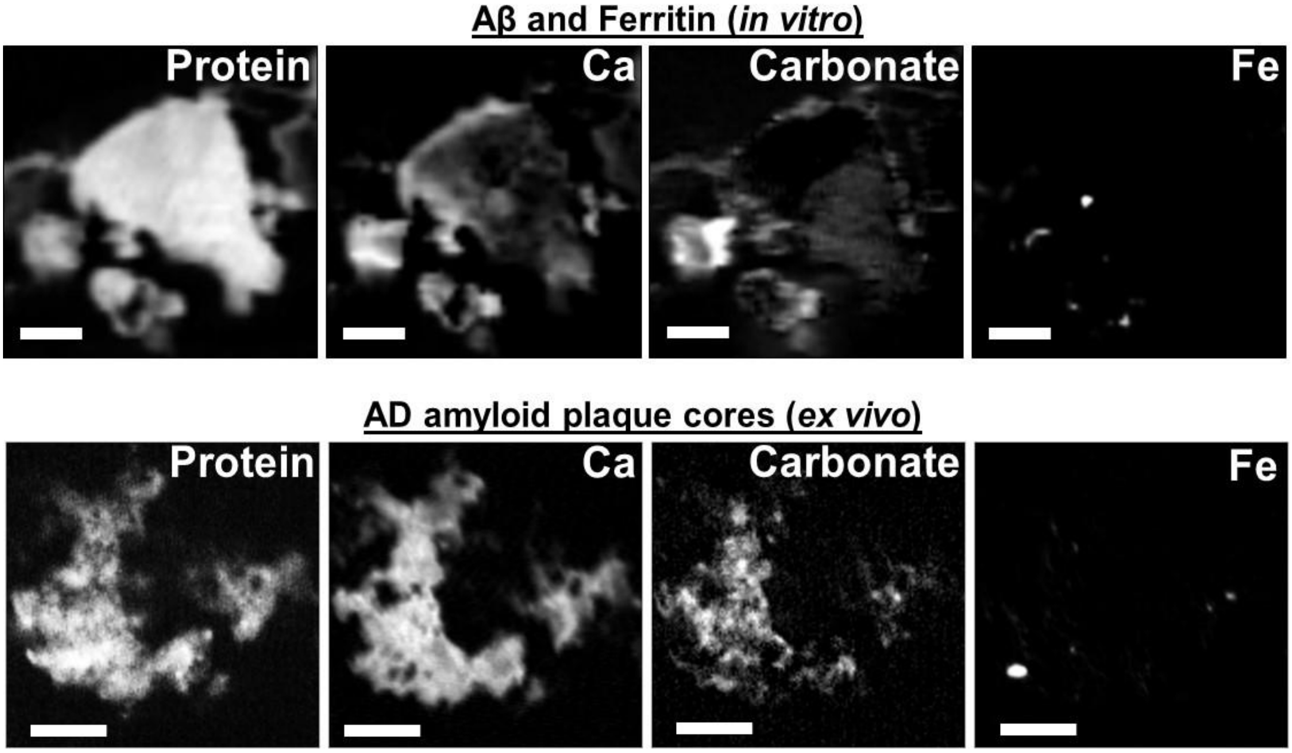

Figure S5. Carbon *K*-edge protein and carbonate maps, calcium *L*-edge maps and Iron L_3-edge maps comparing the Aβ/ferritin aggregate from Figure 6 in the main text (top row), and a human amyloid plaque core from on AD subject (bottom row; from Everett *et al.* 2018 [2]). Scale bars = 2 µm.

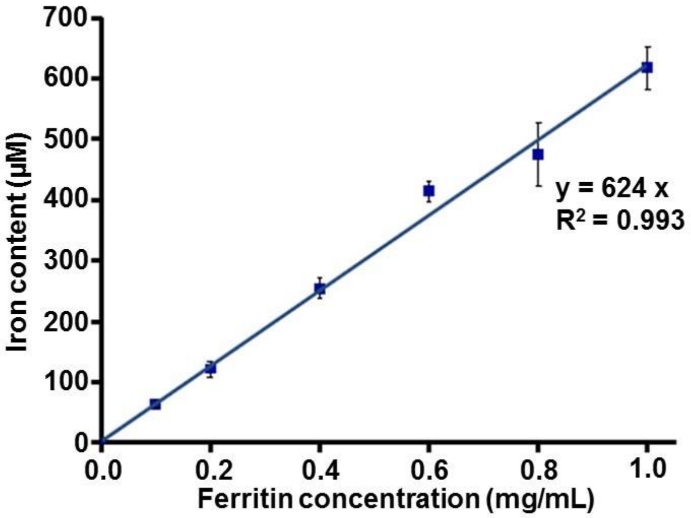

Figure S6. Spectrophotometric determination of ferritin iron content. Spectrophotometric determination of the iron content of ferritin was achieved through a Ferrozine iron quantification assay. Ferrozine selectively binds to Fe^{2+} ions creating a stable magenta coloured solution upon binding, which absorbs light at a wavelength of 562 nm. Ferritin stock (125 mg/mL) was diluted in dH_2O to achieve ferritin concentrations varying between 0.1-1.0 mg/mL. These colloidal ferritin dispersions were acid digested in 2M HNO_3 at 60°C overnight, before being chemically-reduced in 3.3 M hydroxylamine hydrochloride for 3 h at room temperature. 100 µL aliquots of digested/reduced samples were added to 700 µL of 2 mM Ferrozine, and absorbance read at 562 nm using a BioTek plate reader. Iron concentration as a function of ferritin concentration using this approach was determined as 624 µM (0.035 mg/mL) iron per 1 mg/mL (2.2 µM) of ferritin, equating to *ca.* 300 iron atoms per ferritin unit. This sub-maximal loading of iron within horse spleen ferritin is consistent with previous literature [5].

Incubation of ferritin in KH buffer does not result in ferritin iron leaching

To assess the effect of ferritin incubation in KH buffer on the leaching of iron from the ferritin cage, “free” iron concentration as a percentage of total iron content was determined for ferritin suspensions incubated in KH buffer over a 240 hour period.

Briefly, ferritin stock suspensions (from horse spleen, Type I; 125 mg/mL; 1% saline solution; Sigma Aldrich) were diluted dropwise into a 36 mM nitrilotriacetic acid (NTA) solution (pH 7.4), to achieve a final ferritin concentration corresponding to 18 mM iron content (i.e. 2:1 NTA to iron ratio). This step was performed to complex any free iron not bound within the ferritin proteins.

NTA-ferritin suspensions were then diluted in KH buffer (pH 7.4) to the working concentrations described in the main text, and incubated at 37 °C. Samples were taken 0, 48, 144 and 240 hours after the addition of the NTA-ferritin suspension to the KH buffer.

An additional ferritin control sample was prepared by diluting the NTA-ferritin suspensions in dH_2O, as to provide a free iron concentration arising from the starting ferritin suspension.

To assess the level of free iron in the NTA-ferritin suspensions, as a percentage of total iron content, NTA-ferritin suspensions were added to a centrifugal concentrator (Vivaspin 500; 5K MWCO) and spun at 14500 rpm for 40 minutes. The iron concentration of resulting filtrate (containing free iron complexed to NTA, but devoid of intact ferritin), and the starting (unfiltered) NTA-ferritin suspensions were then determined using the Ferrozine iron quantification assay described above.

Free iron concentrations as a percentage of total iron for the NTA-ferritin suspensions are shown in Table S3 and Figure S7. For the NTA-ferritin control suspensions in dH_2O, free iron was shown to account for *ca*. 1.4% of the total iron concentration (±1.5; n=6), demonstrating a low level of free iron and/or disrupted ferritin proteins (thereby allowing NTA to complex with iron) to be present in the starting ferritin suspension. The free iron concentration for NTA-ferritin suspensions

incubated in KH buffer fell within the control sample range for all time points examined (Table S3 and Figure S7; sample size n=3). These results demonstrate that incubation of ferritin in the KH buffer does not result in the leaching of ferritin iron.

Table S3. Free iron concentration as a percentage of total iron for NTA-ferritin suspensions in dH_2O (n=6), and KH buffer (n=3).

**Sample**	**Free iron (% total iron)**	**Standard deviation**
Ferritin in dH_2O	1.37	1.46
**Ferritin in KH buffer incubation series**		
**Incubation time (hrs)**	**Free iron (% total iron)**	**Standard deviation**
0	0.22	0.38
48	0.57	0.57
144	1.65	1.15
240	0.92	0.28

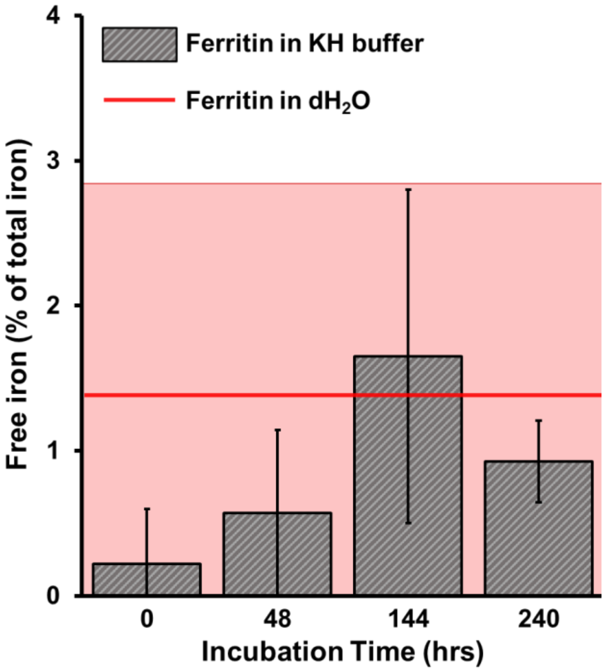

Figure S7. Free iron concentration as a percentage of total iron for NTA-ferritin suspensions, following incubation in KH buffer over a 240 hour time series. Error bars show standard deviation (n=3). The red line shows the free iron concentration recorded in the control NTA-ferritin suspensions in dH_2O (not measured as a function of time). This value corresponds to the free iron present in the starting ferritin solution. The shaded red area shows the standard deviation of these control suspension measurements (n=6).

Supplementary Information References

1. Drits, V. A., Sakharov, B. A., Salyn, A. L. & Manceau, A. Structural model for ferrihydrite. *Clay Minerals.* **28**, 185-207 (1993).
2. Everett, J. *et al.* Nanoscale synchrotron X-ray speciation of iron and calcium compounds in amyloid plaque cores from Alzheimer's disease subjects. *Nanoscale.* **10**, 11782-11796 (2018).
3. Everett, J. *et al.* Ferrous iron formation following the co-aggregation of ferric iron and the Alzheimer's disease peptide beta-amyloid (1-42). *Journal of the Royal Society Interface.* **11**, 20140165 (2014).
4. Everett, J. *et al.* Evidence of Redox-Active Iron Formation Following Aggregation of Ferrihydrite and the Alzheimer's Disease Peptide beta-Amyloid. *Inorganic Chemistry.* **53**, 2803-2809 (2014).
5. Skinner, O. S. *et al.* Native Electron Capture Dissociation Maps to Iron-Binding Channels in Horse Spleen Ferritin. *Analytical Chemistry.* **89**, 10711-10716 (2017).
